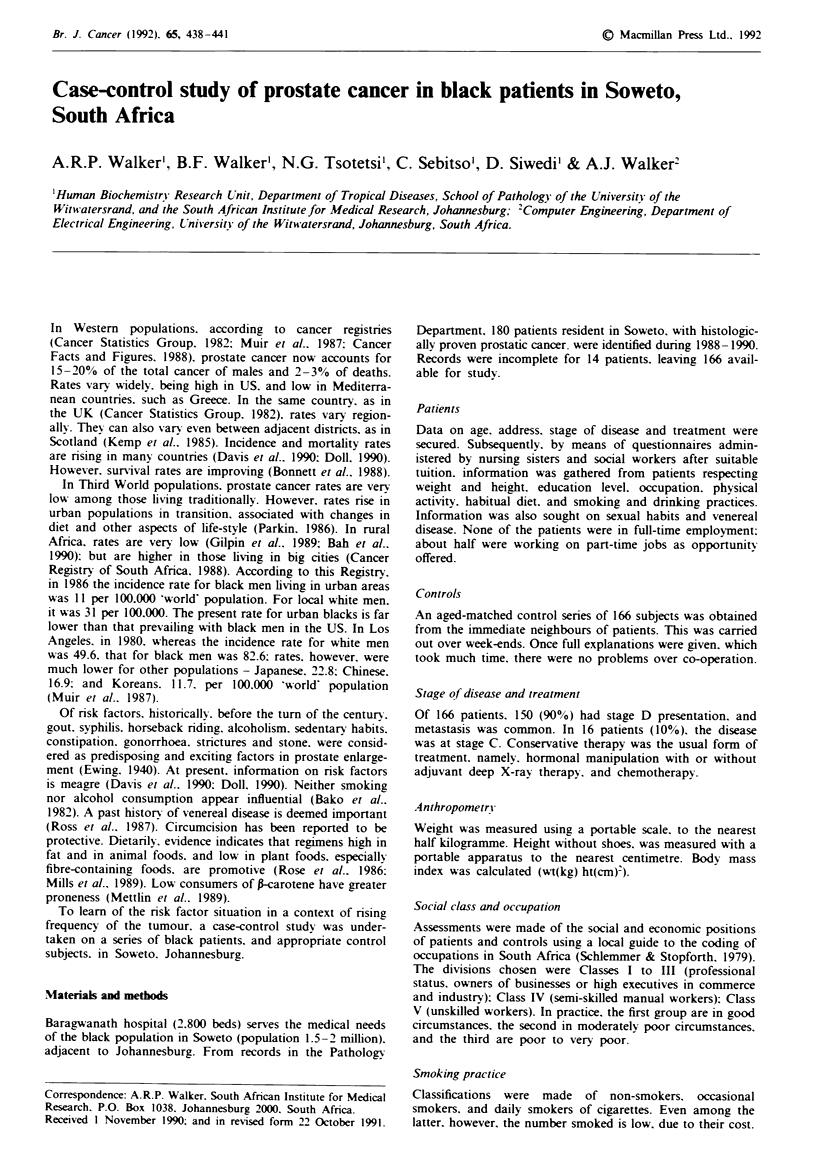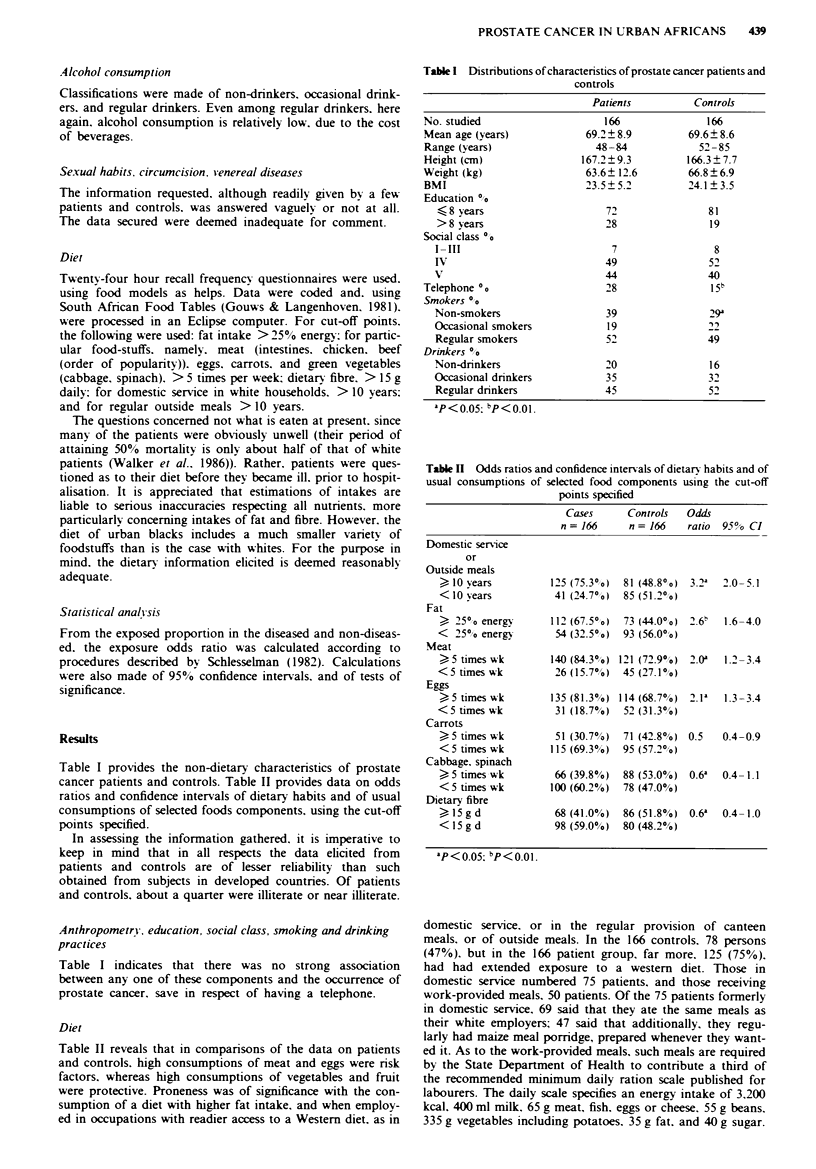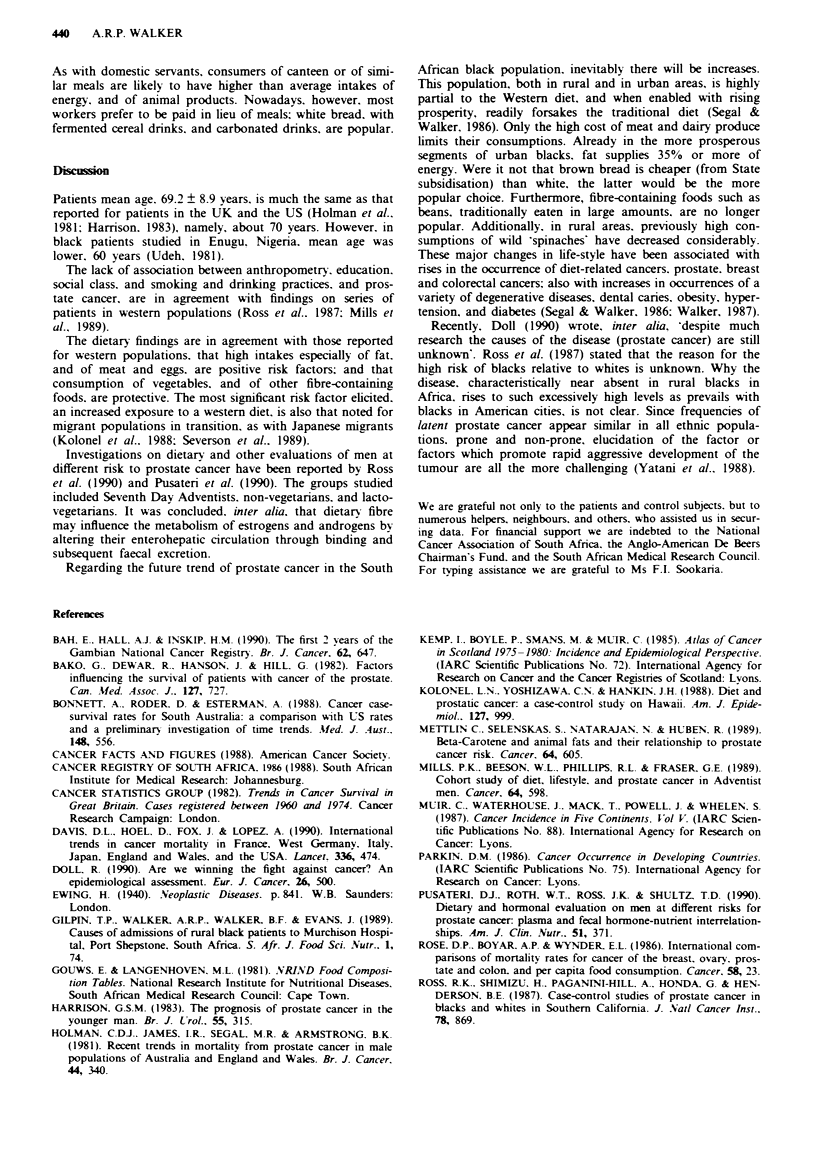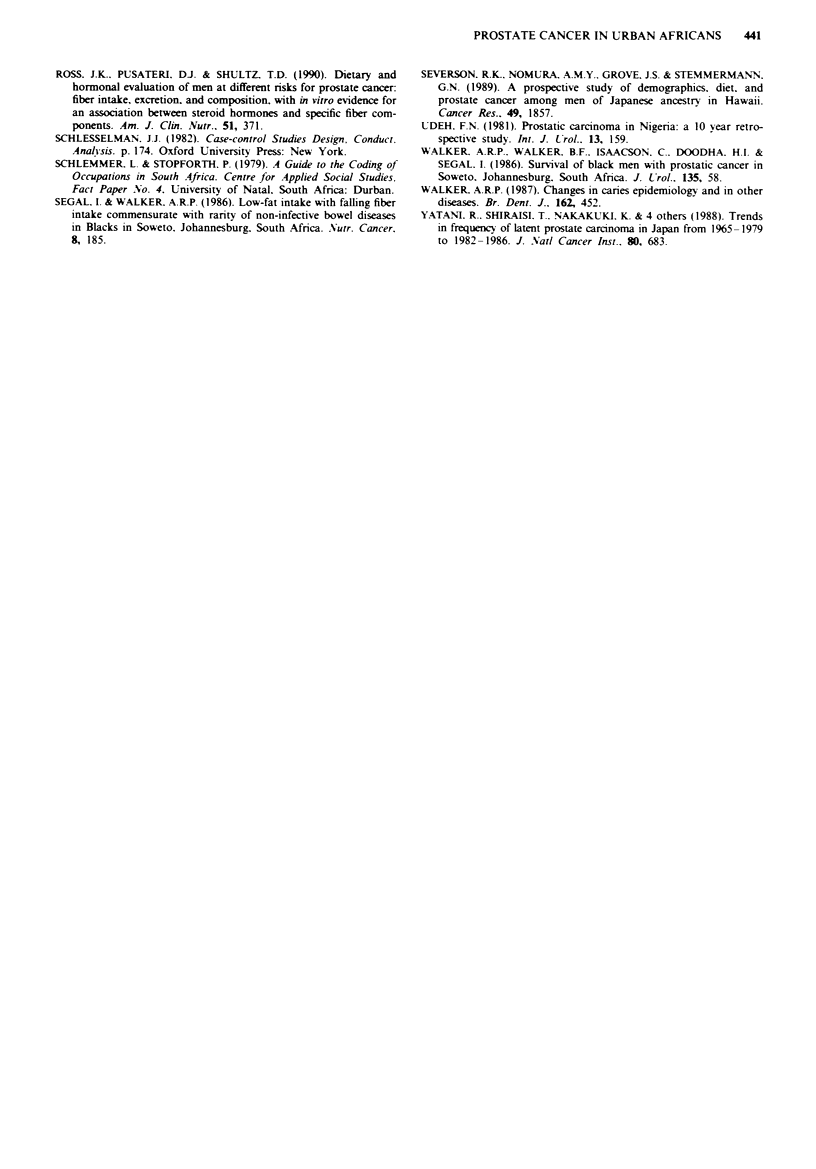# Case-control study of prostate cancer in black patients in Soweto, South Africa.

**DOI:** 10.1038/bjc.1992.89

**Published:** 1992-03

**Authors:** A. R. Walker, B. F. Walker, N. G. Tsotetsi, C. Sebitso, D. Siwedi, A. J. Walker

**Affiliations:** Department of Tropical Diseases, School of Pathology, University of the Witwatersrand, Johannesburg, South Africa.


Br. J. Cancer (1992), 65, 438-441 © Macmillan Press Ltd., 1992

### Case-control study of prostate cancer in black patients in Soweto, South Africa

A.R.P. Walker[1], B.F. Walker[1], N.G. Tsotetsi[1], C. Sebitso[1], D. Siwedi[1] & A.J. Walker[2]

[1]*Human Biochemistry Research Unit, Department of Tropical Diseases, School of Pathology of the University of the Witwatersrand, and the South African Institute for Medical Research, Johannesburg;* [2]*Computer Engineering, Department of Electrical Engineering, University of the Witwatersrand, Johannesburg, South Africa.*

In Western populations, according to cancer registries (Cancer Statistics Group, 1982; Muir *et al.*, 1987; Cancer Facts and Figures, 1988), prostate cancer now accounts for 15–20% of the total cancer of males and 2–3% of deaths. Rates vary widely, being high in US, and low in Mediterranean countries, such as Greece. In the same country, as in the UK (Cancer Statistics Group, 1982), rates vary regionally. They can also vary even between adjacent districts, as in Scotland (Kemp *et al.*, 1985). Incidence and mortality rates are rising in many countries (Davis *et al.*, 1990; Doll, 1990). However, survival rates are improving (Bonnett *et al.*, 1988).

In Third World populations, prostate cancer rates are very low among those living traditionally. However, rates rise in urban populations in transition, associated with changes in diet and other aspects of life-style (Parkin, 1986). In rural Africa, rates are very low (Gilpin *et al.*, 1989; Bah *et al.*, 1990); but are higher in those living in big cities (Cancer Registry of South Africa, 1988). According to this Registry, in 1986 the incidence rate for black men living in urban areas was 11 per 100,000 'world' population. For local white men, it was 31 per 100,000. The present rate for urban blacks is far lower than that prevailing with black men in the US. In Los Angeles, in 1980, whereas the incidence rate for white men was 49.6, that for black men was 82.6; rates, however, were much lower for other populations – Japanese, 22.8; Chinese, 16.9; and Koreans, 11.7, per 100,000 'world' population (Muir *et al.*, 1987).

Of risk factors, historically, before the turn of the century, gout, syphilis, horseback riding, alcoholism, sedentary habits, constipation, gonorrhoea, strictures and stone, were considered as predisposing and exciting factors in prostate enlargement (Ewing, 1940). At present, information on risk factors is meagre (Davis *et al.*, 1990; Doll, 1990). Neither smoking nor alcohol consumption appear influential (Bako *et al.*, 1982). A past history of venereal disease is deemed important (Ross *et al.*, 1987). Circumcision has been reported to be protective. Dietarily, evidence indicates that regimens high in fat and in animal foods, and low in plant foods, especially fibre-containing foods, are promotive (Rose *et al.*, 1986; Mills *et al.*, 1989). Low consumers of β-carotene have greater proneness (Mettlin *et al.*, 1989).

To learn of the risk factor situation in a context of rising frequency of the tumour, a case-control study was undertaken on a series of black patients, and appropriate control subjects, in Soweto, Johannesburg.

### Materials and methods

Baragwanath hospital (2,800 beds) serves the medical needs of the black population in Soweto (population 1.5–2 million), adjacent to Johannesburg. From records in the Pathology Department, 180 patients resident in Soweto, with histologically proven prostatic cancer, were identified during 1988–1990. Records were incomplete for 14 patients, leaving 166 available for study.

### *Patients*

Data on age, address, stage of disease and treatment were secured. Subsequently, by means of questionnaires administered by nursing sisters and social workers after suitable tuition, information was gathered from patients respecting weight and height, education level, occupation, physical activity, habitual diet, and smoking and drinking practices. Information was also sought on sexual habits and venereal disease. None of the patients were in full-time employment; about half were working on part-time jobs as opportunity offered.

### *Controls*

An aged-matched control series of 166 subjects was obtained from the immediate neighbours of patients. This was carried out over week-ends. Once full explanations were given, which took much time, there were no problems over co-operation.

### *Stage of disease and treatment*

Of 166 patients, 150 (90%) had stage D presentation, and metastasis was common. In 16 patients (10%), the disease was at stage C. Conservative therapy was the usual form of treatment, namely, hormonal manipulation with or without adjuvant deep X-ray therapy, and chemotherapy.

### *Anthropometry*

Weight was measured using a portable scale, to the nearest half kilogramme. Height without shoes, was measured with a portable apparatus to the nearest centimetre. Body mass index was calculated (wt(kg) ht(cm)$^2$).

### *Social class and occupation*

Assessments were made of the social and economic positions of patients and controls using a local guide to the coding of occupations in South Africa (Schlemmer & Stopforth, 1979). The divisions chosen were Classes I to III (professional status, owners of businesses or high executives in commerce and industry); Class IV (semi-skilled manual workers); Class V (unskilled workers). In practice, the first group are in good circumstances, the second in moderately poor circumstances, and the third are poor to very poor.

### *Smoking practice*

Classifications were made of non-smokers, occasional smokers, and daily smokers of cigarettes. Even among the latter, however, the number smoked is low, due to their cost.

Correspondence: A.R.P. Walker, South African Institute for Medical Research, P.O. Box 1038, Johannesburg 2000, South Africa.
Received 1 November 1990; and in revised form 22 October 1991.

*Alcohol consumption*

Classifications were made of non-drinkers, occasional drinkers, and regular drinkers. Even among regular drinkers, here again, alcohol consumption is relatively low, due to the cost of beverages.

*Sexual habits, circumcision, venereal diseases*

The information requested, although readily given by a few patients and controls, was answered vaguely or not at all. The data secured were deemed inadequate for comment.

*Diet*

Twenty-four hour recall frequency questionnaires were used, using food models as helps. Data were coded and, using South African Food Tables (Gouws & Langenhoven, 1981), were processed in an Eclipse computer. For cut-off points, the following were used: fat intake >25% energy; for particular food-stuffs, namely, meat (intestines, chicken, beef (order of popularity)), eggs, carrots, and green vegetables (cabbage, spinach), >5 times per week; dietary fibre, >15 g daily; for domestic service in white households, >10 years; and for regular outside meals >10 years.

The questions concerned not what is eaten at present, since many of the patients were obviously unwell (their period of attaining 50% mortality is only about half of that of white patients (Walker *et al*., 1986)). Rather, patients were questioned as to their diet before they became ill, prior to hospitalisation. It is appreciated that estimations of intakes are liable to serious inaccuracies respecting all nutrients, more particularly concerning intakes of fat and fibre. However, the diet of urban blacks includes a much smaller variety of foodstuffs than is the case with whites. For the purpose in mind, the dietary information elicited is deemed reasonably adequate.

*Statistical analysis*

From the exposed proportion in the diseased and non-diseased, the exposure odds ratio was calculated according to procedures described by Schlesselman (1982). Calculations were also made of 95% confidence intervals, and of tests of significance.

### Results

Table I provides the non-dietary characteristics of prostate cancer patients and controls. Table II provides data on odds ratios and confidence intervals of dietary habits and of usual consumptions of selected foods components, using the cut-off points specified.

In assessing the information gathered, it is imperative to keep in mind that in all respects the data elicited from patients and controls are of lesser reliability than such obtained from subjects in developed countries. Of patients and controls, about a quarter were illiterate or near illiterate.

*Anthropometry, education, social class, smoking and drinking practices*

Table I indicates that there was no strong association between any one of these components and the occurrence of prostate cancer, save in respect of having a telephone.

*Diet*

Table II reveals that in comparisons of the data on patients and controls, high consumptions of meat and eggs were risk factors, whereas high consumptions of vegetables and fruit were protective. Proneness was of significance with the consumption of a diet with higher fat intake, and when employed in occupations with readier access to a Western diet, as in domestic service, or in the regular provision of canteen meals, or of outside meals. In the 166 controls, 78 persons (47%), but in the 166 patient group, far more, 125 (75%), had had extended exposure to a western diet. Those in domestic service numbered 75 patients, and those receiving work-provided meals, 50 patients. Of the 75 patients formerly in domestic service, 69 said that they ate the same meals as their white employers; 47 said that additionally, they regularly had maize meal porridge, prepared whenever they wanted it. As to the work-provided meals, such meals are required by the State Department of Health to contribute a third of the recommended minimum daily ration scale published for labourers. The daily scale specifies an energy intake of 3,200 kcal, 400 ml milk, 65 g meat, fish, eggs or cheese, 55 g beans, 335 g vegetables including potatoes, 35 g fat, and 40 g sugar.

**Table I** Distributions of characteristics of prostate cancer patients and controls

| | *Patients* | *Controls* |
|---|---|---|
| No. studied | 166 | 166 |
| Mean age (years) | 69.2±8.9 | 69.6±8.6 |
| Range (years) | 48–84 | 52–85 |
| Height (cm) | 167.2±9.3 | 166.3±7.7 |
| Weight (kg) | 63.6±12.6 | 66.8±6.9 |
| BMI | 23.5±5.2 | 24.1±3.5 |
| Education % | | |
| ≤8 years | 72 | 81 |
| >8 years | 28 | 19 |
| Social class % | | |
| I–III | 7 | 8 |
| IV | 49 | 52 |
| V | 44 | 40 |
| Telephone % | 28 | 15[b] |
| *Smokers %* | | |
| Non-smokers | 39 | 29[a] |
| Occasional smokers | 19 | 22 |
| Regular smokers | 52 | 49 |
| *Drinkers %* | | |
| Non-drinkers | 20 | 16 |
| Occasional drinkers | 35 | 32 |
| Regular drinkers | 45 | 52 |

[a]$P<0.05$; [b]$P<0.01$.

**Table II** Odds ratios and confidence intervals of dietary habits and of usual consumptions of selected food components using the cut-off points specified

| | *Cases* $n=166$ | *Controls* $n=166$ | *Odds ratio* | *95% CI* |
|---|---|---|---|---|
| Domestic service or Outside meals | | | | |
| ≥10 years | 125 (75.3%) | 81 (48.8%) | 3.2[a] | 2.0–5.1 |
| <10 years | 41 (24.7%) | 85 (51.2%) | | |
| Fat | | | | |
| ≥ 25% energy | 112 (67.5%) | 73 (44.0%) | 2.6[b] | 1.6–4.0 |
| < 25% energy | 54 (32.5%) | 93 (56.0%) | | |
| Meat | | | | |
| ≥5 times wk | 140 (84.3%) | 121 (72.9%) | 2.0[a] | 1.2–3.4 |
| <5 times wk | 26 (15.7%) | 45 (27.1%) | | |
| Eggs | | | | |
| ≥5 times wk | 135 (81.3%) | 114 (68.7%) | 2.1[a] | 1.3–3.4 |
| <5 times wk | 31 (18.7%) | 52 (31.3%) | | |
| Carrots | | | | |
| ≥5 times wk | 51 (30.7%) | 71 (42.8%) | 0.5 | 0.4–0.9 |
| <5 times wk | 115 (69.3%) | 95 (57.2%) | | |
| Cabbage, spinach | | | | |
| ≥5 times wk | 66 (39.8%) | 88 (53.0%) | 0.6[a] | 0.4–1.1 |
| <5 times wk | 100 (60.2%) | 78 (47.0%) | | |
| Dietary fibre | | | | |
| ≥15 g d | 68 (41.0%) | 86 (51.8%) | 0.6[a] | 0.4–1.0 |
| <15 g d | 98 (59.0%) | 80 (48.2%) | | |

[a]$P<0.05$; [b]$P<0.01$.

As with domestic servants, consumers of canteen or of similar meals are likely to have higher than average intakes of energy, and of animal products. Nowadays, however, most workers prefer to be paid in lieu of meals; white bread, with fermented cereal drinks, and carbonated drinks, are popular.

### Discussion

Patients mean age, 69.2 ± 8.9 years, is much the same as that reported for patients in the UK and the US (Holman *et al.*, 1981; Harrison, 1983), namely, about 70 years. However, in black patients studied in Enugu, Nigeria, mean age was lower, 60 years (Udeh, 1981).

The lack of association between anthropometry, education, social class, and smoking and drinking practices, and prostate cancer, are in agreement with findings on series of patients in western populations (Ross *et al.*, 1987; Mills *et al.*, 1989).

The dietary findings are in agreement with those reported for western populations, that high intakes especially of fat, and of meat and eggs, are positive risk factors; and that consumption of vegetables, and of other fibre-containing foods, are protective. The most significant risk factor elicited, an increased exposure to a western diet, is also that noted for migrant populations in transition, as with Japanese migrants (Kolonel *et al.*, 1988; Severson *et al.*, 1989).

Investigations on dietary and other evaluations of men at different risk to prostate cancer have been reported by Ross *et al.* (1990) and Pusateri *et al.* (1990). The groups studied included Seventh Day Adventists, non-vegetarians, and lacto-vegetarians. It was concluded, *inter alia*, that dietary fibre may influence the metabolism of estrogens and androgens by altering their enterohepatic circulation through binding and subsequent faecal excretion.

Regarding the future trend of prostate cancer in the South African black population, inevitably there will be increases. This population, both in rural and in urban areas, is highly partial to the Western diet, and when enabled with rising prosperity, readily forsakes the traditional diet (Segal & Walker, 1986). Only the high cost of meat and dairy produce limits their consumptions. Already in the more prosperous segments of urban blacks, fat supplies 35% or more of energy. Were it not that brown bread is cheaper (from State subsidisation) than white, the latter would be the more popular choice. Furthermore, fibre-containing foods such as beans, traditionally eaten in large amounts, are no longer popular. Additionally, in rural areas, previously high consumptions of wild 'spinaches' have decreased considerably. These major changes in life-style have been associated with rises in the occurrence of diet-related cancers, prostate, breast and colorectal cancers; also with increases in occurrences of a variety of degenerative diseases, dental caries, obesity, hypertension, and diabetes (Segal & Walker, 1986; Walker, 1987).

Recently, Doll (1990) wrote, *inter alia*, 'despite much research the causes of the disease (prostate cancer) are still unknown'. Ross *et al.* (1987) stated that the reason for the high risk of blacks relative to whites is unknown. Why the disease, characteristically near absent in rural blacks in Africa, rises to such excessively high levels as prevails with blacks in American cities, is not clear. Since frequencies of *latent* prostate cancer appear similar in all ethnic populations, prone and non-prone, elucidation of the factor or factors which promote rapid aggressive development of the tumour are all the more challenging (Yatani *et al.*, 1988).

We are grateful not only to the patients and control subjects, but to numerous helpers, neighbours, and others, who assisted us in securing data. For financial support we are indebted to the National Cancer Association of South Africa, the Anglo-American De Beers Chairman's Fund, and the South African Medical Research Council. For typing assistance we are grateful to Ms F.I. Sookaria.

### References

BAH, E., HALL, A.J. & INSKIP, H.M. (1990). The first 2 years of the Gambian National Cancer Registry. *Br. J. Cancer*, **62**, 647.

BAKO, G., DEWAR, R., HANSON, J. & HILL, G. (1982). Factors influencing the survival of patients with cancer of the prostate. *Can. Med. Assoc. J.*, **127**, 727.

BONNETT, A., RODER, D. & ESTERMAN, A. (1988). Cancer case-survival rates for South Australia: a comparison with US rates and a preliminary investigation of time trends. *Med. J. Aust.*, **148**, 556.

CANCER FACTS AND FIGURES (1988). American Cancer Society.

CANCER REGISTRY OF SOUTH AFRICA, 1986 (1988). South African Institute for Medical Research: Johannesburg.

CANCER STATISTICS GROUP (1982). *Trends in Cancer Survival in Great Britain. Cases registered between 1960 and 1974*. Cancer Research Campaign: London.

DAVIS, D.L., HOEL, D., FOX, J. & LOPEZ, A. (1990). International trends in cancer mortality in France, West Germany, Italy, Japan, England and Wales, and the USA. *Lancet*, **336**, 474.

DOLL, R. (1990). Are we winning the fight against cancer? An epidemiological assessment. *Eur. J. Cancer*, **26**, 500.

EWING, H. (1940). *Neoplastic Diseases*. p. 841. W.B. Saunders: London.

GILPIN, T.P., WALKER, A.R.P., WALKER, B.F. & EVANS, J. (1989). Causes of admissions of rural black patients to Murchison Hospital, Port Shepstone, South Africa. *S. Afr. J. Food Sci. Nutr.*, **1**, 74.

GOUWS, E. & LANGENHOVEN, M.L. (1981). *NRIND Food Composition Tables*. National Research Institute for Nutritional Diseases, South African Medical Research Council: Cape Town.

HARRISON, G.S.M. (1983). The prognosis of prostate cancer in the younger man. *Br. J. Urol.*, **55**, 315.

HOLMAN, C.D.J., JAMES, I.R., SEGAL, M.R. & ARMSTRONG, B.K. (1981). Recent trends in mortality from prostate cancer in male populations of Australia and England and Wales. *Br. J. Cancer*, **44**, 340.

KEMP, I., BOYLE, P., SMANS, M. & MUIR, C. (1985). *Atlas of Cancer in Scotland 1975–1980: Incidence and Epidemiological Perspective*. (IARC Scientific Publications No. 72). International Agency for Research on Cancer and the Cancer Registries of Scotland: Lyons.

KOLONEL, L.N., YOSHIZAWA, C.N. & HANKIN, J.H. (1988). Diet and prostatic cancer: a case-control study on Hawaii. *Am. J. Epidemiol.*, **127**, 999.

METTLIN C., SELENSKAS, S., NATARAJAN, N. & HUBEN, R. (1989). Beta-Carotene and animal fats and their relationship to prostate cancer risk. *Cancer*, **64**, 605.

MILLS, P.K., BEESON, W.L., PHILLIPS, R.L. & FRASER, G.E. (1989). Cohort study of diet, lifestyle, and prostate cancer in Adventist men. *Cancer*, **64**, 598.

MUIR, C., WATERHOUSE, J., MACK, T., POWELL, J. & WHELEN, S. (1987). *Cancer Incidence in Five Continents, Vol V*. (IARC Scientific Publications No. 88). International Agency for Research on Cancer: Lyons.

PARKIN, D.M. (1986). *Cancer Occurrence in Developing Countries*. (IARC Scientific Publications No. 75). International Agency for Research on Cancer: Lyons.

PUSATERI, D.J., ROTH, W.T., ROSS, J.K. & SHULTZ, T.D. (1990). Dietary and hormonal evaluation on men at different risks for prostate cancer: plasma and fecal hormone-nutrient interrelationships. *Am. J. Clin. Nutr.*, **51**, 371.

ROSE, D.P., BOYAR, A.P. & WYNDER, E.L. (1986). International comparisons of mortality rates for cancer of the breast, ovary, prostate and colon, and per capita food consumption. *Cancer*, **58**, 23.

ROSS, R.K., SHIMIZU, H., PAGANINI-HILL, A., HONDA, G. & HENDERSON, B.E. (1987). Case-control studies of prostate cancer in blacks and whites in Southern California. *J. Natl Cancer Inst.*, **78**, 869.

ROSS, J.K., PUSATERI, D.J. & SHULTZ, T.D. (1990). Dietary and hormonal evaluation of men at different risks for prostate cancer: fiber intake, excretion, and composition, with *in vitro* evidence for an association between steroid hormones and specific fiber components. *Am. J. Clin. Nutr.*, **51**, 371.

SCHLESSELMAN, J.J. (1982). *Case-control Studies Design, Conduct, Analysis*. p. 174. Oxford University Press: New York.

SCHLEMMER, L. & STOPFORTH, P. (1979). *A Guide to the Coding of Occupations in South Africa. Centre for Applied Social Studies, Fact Paper No. 4*. University of Natal, South Africa: Durban.

SEGAL, I. & WALKER, A.R.P. (1986). Low-fat intake with falling fiber intake commensurate with rarity of non-infective bowel diseases in Blacks in Soweto, Johannesburg, South Africa. *Nutr. Cancer*, **8**, 185.

SEVERSON, R.K., NOMURA, A.M.Y., GROVE, J.S. & STEMMERMANN, G.N. (1989). A prospective study of demographics, diet, and prostate cancer among men of Japanese ancestry in Hawaii. *Cancer Res.*, **49**, 1857.

UDEH, F.N. (1981). Prostatic carcinoma in Nigeria: a 10 year retrospective study. *Int. J. Urol.*, **13**, 159.

WALKER, A.R.P., WALKER, B.F., ISAACSON, C., DOODHA, H.I. & SEGAL, I. (1986). Survival of black men with prostatic cancer in Soweto, Johannesburg, South Africa. *J. Urol.*, **135**, 58.

WALKER, A.R.P. (1987). Changes in caries epidemiology and in other diseases. *Br. Dent. J.*, **162**, 452.

YATANI, R., SHIRAISI, T., NAKAKUKI, K. & 4 others (1988). Trends in frequency of latent prostate carcinoma in Japan from 1965–1979 to 1982–1986. *J. Natl Cancer Inst.*, **80**, 683.